# An Uncommon Presentation of a Ruptured Tubal Ectopic Pregnancy With Broad Ligament Hematoma

**DOI:** 10.7759/cureus.81605

**Published:** 2025-04-02

**Authors:** Ritu Singh, Deeptimayee Pathi, Priyanka, Diksha

**Affiliations:** 1 Obstetrics and Gynecology, Kurji Holy Family Hospital, Patna, IND

**Keywords:** broad ligament hematoma, ectopic pregnancy, internal iliac artery ligation, pregnancy, ruptured ectopic pregnancy, surgical case report, uncommon presentation

## Abstract

Broad ligament hematomas are rare in obstetrics. These hematomas typically result from vaginal, cervical, or uterine tears that extend to the uterine or vaginal arteries. Although cases of intestinal ectopic pregnancies presenting as broad ligament hematomas with no intraperitoneal collection have been reported, no cases involving broad ligament hematomas along with hemoperitoneum following ampullary ruptured ectopic pregnancy have been documented. Internal iliac artery ligation (IIAL) is particularly useful in broad ligament hematomas. So, we present a case of ruptured ampullary ectopic pregnancy with both intraperitoneal collection and broad ligament hematoma managed by salpingectomy and bilateral IIAL.

## Introduction

Broad ligament hematomas are rare in obstetrics, occurring in approximately one in 20,000 cases [1], and may be caused by operative vaginal or cesarean deliveries or may occur spontaneously due to the rupture of uterine arteries or varices. These hematomas typically result from vaginal, cervical, or uterine tears that extend to the uterine or vaginal arteries [2].

Spontaneous uterine artery rupture, which causes broad ligament hematomas, is associated with a 40% maternal mortality rate when occurring during labor, possibly due to blood pressure fluctuations during labor [3]. Hormonal changes during pregnancy, especially increased estrogen levels, are believed to play a role by suppressing intimal proliferation in response to vascular injury, making vessels more prone to rupture [4]. Although cases of intestinal ectopic pregnancies presenting as broad ligament hematomas with no intraperitoneal collection have been reported [5], there is also a case report of ovarian ectopic pregnancy presenting as hemoperitoneum and broad ligament hematoma [6], but no cases involving broad ligament hematomas along with hemoperitoneum following ampullary ruptured ectopic pregnancy have been documented.

Internal iliac artery ligation (IIAL) is particularly useful in broad ligament hematomas when torn vessels retract within the broad ligament [7].

So, we present a case of a ruptured ampullary ectopic pregnancy with both an intraperitoneal collection and a broad ligament hematoma, managed by salpingectomy and bilateral IIAL, after obtaining informed consent from the patient for publication.

## Case presentation

A 34-year-old female presented to the emergency department with 5-7 days of abdominal pain, discomfort, nausea, and vomiting. The pain became severe, prompting her to seek medical attention. She denied any history of amenorrhea or abnormal vaginal bleeding. Her last menstrual period was 15 days ago, and her cycles were regular. She had two live children. The first was delivered vaginally 14 years ago and the second by cesarean section 4.5 years ago. On examination, her vitals showed pallor, tachycardia (114/min), hypotension (BP 90/50 mmHg), dry tongue, and oxygen saturation was 99% on room air. Abdominal examination revealed mild tenderness. Her vaginal examination revealed a normal-sized, anteverted uterus with cervical motion tenderness. Investigations were done as shown in Table 1.

**Table 1:** Laboratory parameters INR: international normalized ratio

Lab parameter	Lab values	Reference range
Hemoglobin	7	12-16 gm/dl
Total leukocyte count	7.81 x 10^3^	5.2-12.4 x 10^3 ^cells/microL
Platelet	140	140-400 x 10^3 ^cells/microL
Serum bilirubin direct/total	0.1/0.5	0.5-1.5/0-0.6 mg/dl
Alanine aminotransferase (ALT)	15	0-40 U/L
Prothrombin time/INR	17/1.31	11-14 sec/0.9-1.3
Random blood sugar	73	75-139 mg/dl
Urea	27	10-50 mg/dl
Creatinine	0.6	0.6-1.4 mg/dl
Serum sodium	135	135-145 meq/L
Serum potassium	3.5	3.5-5.0 meq/L
Serum chloride	102	98-100 meq/L

**Figure 1:**
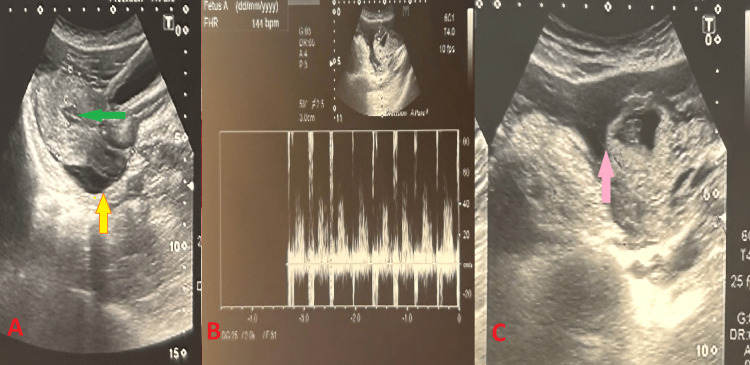
Radiological images (A) The green arrow shows an empty uterus, and the yellow arrow shows a pouch of Douglas collection. (B) shows fetal heart rate. (C) The pink arrow shows hemoperitoneum.

She had hemoglobin of 7 gm/dl. Ultrasound showed an empty uterus with a pouch of Douglas as shown in Figure 1A.

**Figure 2:**
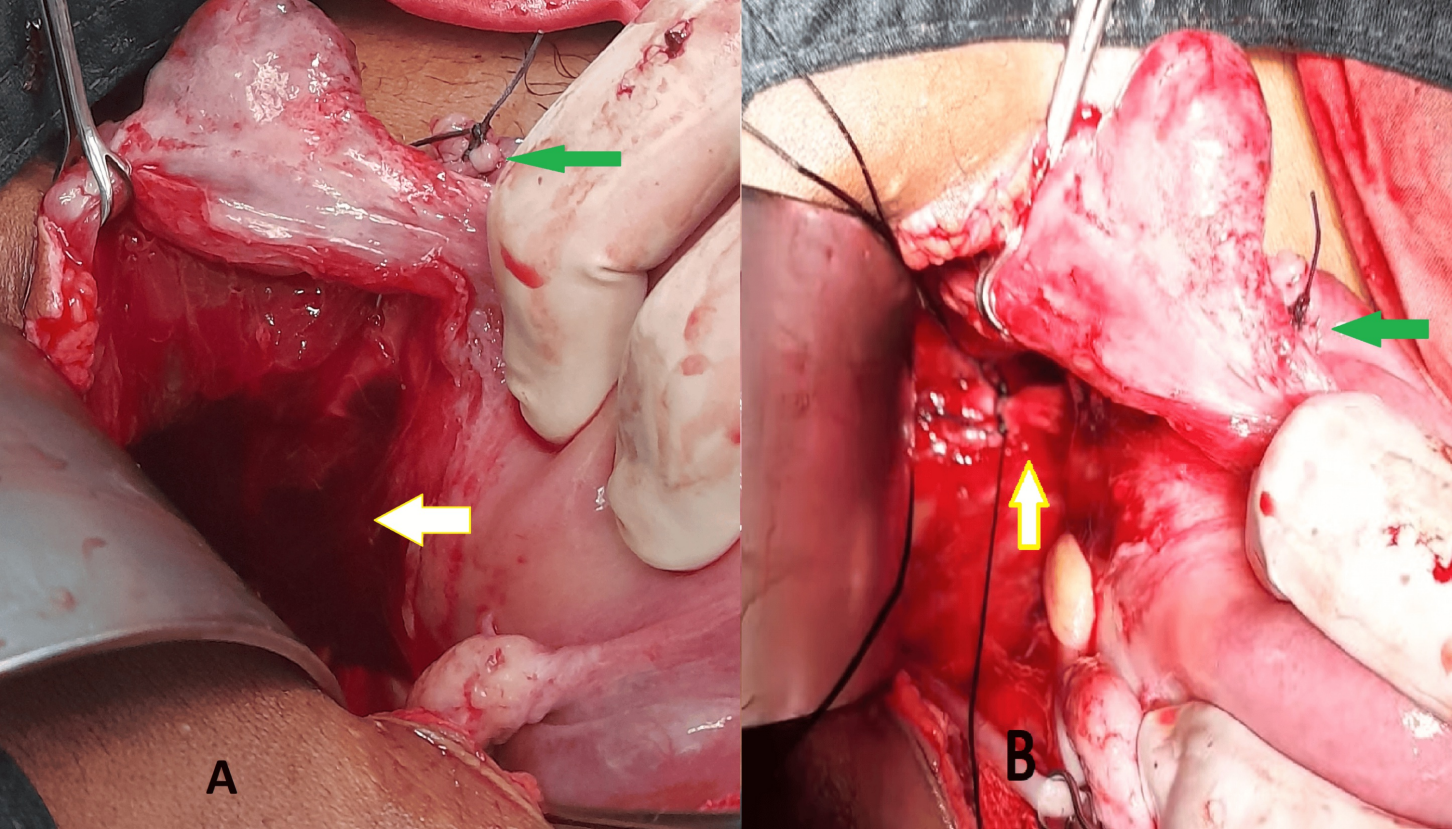
Operative images (A) The white arrow shows a broad ligament hematoma; the green arrow shows the stump of salpingectomy done for ectopic pregnancy. (B) The white arrow shows internal artery ligation; the green arrow shows the stump of salpingectomy done for ectopic pregnancy.

There was a left adnexal gestational sac with a crown-rump length of 7.3 mm, corresponding to six weeks and four days of pregnancy, with cardiac activity as shown in Figure 1B, and there was a hemoperitoneum as shown in Figure 1C. The right ovary was normal. The findings were suggestive of a ruptured left tubal ectopic pregnancy. After the ultrasound, we did her urine pregnancy test and beta-human chorionic gonadotropin (HCG), which came back positive and 35,100 mIU/mL, respectively, as the patient didn’t give a history of amenorrhea. Meanwhile, she received one unit of blood and was posted for exploratory laparotomy with consent for left salpingectomy and right tubal ligation. On opening the abdomen, there was hemoperitoneum of about 1.5 liters. Left side ruptured tubal ectopic pregnancy was noted with fresh bleeding. And to the surprise, there was a broad ligament hematoma below the tube, with smooth surfaces, no rupture, and no obvious active bleeding as shown in Figure 2A.

It was suspected that the hematomas resulted from the spontaneous rupture of the fallopian tube branch of the uterine artery in this region, causing bleeding into the broad ligament. The rest of the abdominal survey did not reveal any other abnormalities. A left salpingectomy was performed (Figure 2), along with right-sided tubal ligation. And the decision of bilateral IIAL was made; as for broad ligament hematoma, IIAL is useful in cases of postoperative hemorrhage when no definitive bleeding point is identifiable. The illustration of IIAL is shown in Figure 2B. Peritoneal washings were done, and a drain was placed. The procedure was well-tolerated, with slightly above-average blood loss, requiring two units of packed RBCs postoperatively. The postoperative period was uneventful, and the drain was removed on postoperative day 2. Her hemoglobin improved to 8.7 g/dL, and she was discharged on postoperative day 4.

The IIAL procedure was performed as follows: A posterior approach to the IIAL was used. The bowel was packed and retracted back. The ureter was grasped with a Babcock tissue-holding forceps, and another Babcock was used to grasp the infundibulopelvic ligament. The sacral promontory was palpated as a landmark, where the bifurcation of the common iliac artery into the internal and external iliac arteries lies. A loose fold of peritoneum, located laterally to the ureter and medially to the infundibulopelvic ligament, was carefully identified. The fold was grasped at two points using artery forceps, ensuring that it remained parallel to the ureter. A small nick was made over the hold of the artery forceps using scissors, allowing the peritoneum to be incised. Once the nick was made, the peritoneum was gently opened with the fingers, and dissection was carried out parallel to the vessels. During this step, the ureter remained attached to the medial fold of the peritoneal reflection, preserving its position and minimizing the risk of damage.

After identifying the internal iliac artery 4 cm away from the bifurcation, the Mixter forceps were inserted from the lateral to the medial side, underneath the internal iliac artery. Suture material was then fed in the form of a loop into the Mixter forceps, and the Mixters drew the suture material below the internal iliac artery. The knot was tied and pushed into place using fingers. The internal iliac artery was ligated doubly, with the ligatures placed 0.5 cm apart. The femoral and dorsalis pedis pulses were palpated to confirm that the internal iliac artery, not the external iliac artery, had been ligated.

## Discussion

Our case describes a 34-year-old female who presented with abdominal pain, discomfort, nausea, and vomiting and was eventually diagnosed with a ruptured left-sided tubal ectopic pregnancy. She did not have any history of amenorrhea or vaginal bleeding, which aligns with the fact that the classic triad of amenorrhea, vaginal bleeding, and pelvic pain is present in fewer than 50% of ectopic pregnancy cases [8]. Amenorrhea is the most common feature of ectopic pregnancy, as found in the study by Nalini et al., where only two out of 75 patients did not have amenorrhea [9]. This makes our case even more unique.

The broad ligament, a double layer of peritoneum supporting the uterus, fallopian tubes, and ovaries, plays a critical role in maintaining the position of reproductive organs. These hematomas usually result from trauma to the pelvic vasculature during instrumental vaginal delivery, cesarean sections, or other pelvic surgeries [10].

But there are many case reports of broad ligament hematoma because of unusual causes. Maqbool et al. reported a complex case of placenta percreta involving the broad ligament and urinary bladder in a twin pregnancy. Despite a timely hysterectomy and arterial ligation, the patient succumbed to a massive hemorrhage [11]. Tokuda et al. discussed a case of uterine perforation after dilation and curettage, which led to a hematoma under the broad ligament, requiring surgical intervention due to clinical deterioration [12]. Kurakula et al. reported a case of uterine rupture in an unscarred uterus, resulting in a broad ligament hematoma discovered during tubal ligation. Surgical treatment was initiated upon clinical deterioration [13]. There was also a case report of a 23-year-old female who developed a broad ligament pregnancy diagnosed intraoperatively during laparoscopic surgery. The patient underwent resection of the lesion and salpingectomy after significant hemorrhage [14]. Bankada et al. described a rare case of bilateral broad ligament hematoma in a twin pregnancy [15]. But to the best of our search, we had not found any case of rupture ectopic pregnancy, which led to both intraperitoneal hemorrhage and broad ligament hematoma as in our case.

Management of broad ligament hematomas depends on the hematoma’s size and the patient’s hemodynamic condition. Small, stable hematomas may be managed conservatively with close monitoring, fluid resuscitation, and pain management. In contrast, larger or expanding hematomas, or those causing significant hemodynamic instability, may require surgical intervention, including hematoma evacuation and vessel ligation [10].

Recognizing and managing broad ligament hematomas is crucial in obstetric practice, as untreated cases can lead to complications such as hypovolemic shock, infection, long-term reproductive issues [10], and death. A case reported by Varvoutis et al. described a 22-year-old woman who developed a broad ligament hematoma post-vaginal delivery with chorioamnionitis and pre-eclampsia. Despite initial treatment and uterine evacuation, the patient required a hysterectomy due to an infected broad ligament hematoma [16].

IIAL is a safe and effective method for controlling bleeding in the genital tract. In life-threatening situations, it can be a valuable alternative to hysterectomy [17]. Bilateral IIAL reduces pelvic blood flow by 49% and pulse pressure by 85%, promoting hemostasis by creating retrograde blood flow in the collaterals, which prevents pelvic ischemia. The procedure is highly effective, with reported success rates ranging from 42% to 100% [18]. Although technically difficult, IIAL is an effective procedure with minimal complications and a short learning curve [19]. One of the main challenges with IIAL is the surgeon's hesitation to perform it early enough [17].

IIAL has been successfully used in various conditions, including atonic PPH, cervical pregnancy, placenta increta, and uterine trauma [17]. In cases of uterine trauma, where it is difficult to locate the avulsed uterine artery due to its retraction into the broad ligament, IIAL helps control hemorrhage and avoid hysterectomy. The procedure also helps create a relatively bloodless operative field, reducing the risk of injury to structures like the ureter [17].

After IIAL, Doppler examinations had shown collateral circulation developing in all cases, and recanalization of the internal iliac artery occurred in 90% of patients at the six-month follow-up, suggesting that future fertility and long-term clinical outcomes are not adversely affected [17].

## Conclusions

Being vigilant for ectopic pregnancy is crucial, as patients may not present with the classic triad of amenorrhea, abdominal pain, and vaginal bleeding. Immediate intervention is essential to manage an acute abdomen.

This case report highlights the rarity and significance of uncommon presentations following a ruptured ectopic pregnancy, underscoring the need for a high level of suspicion and prompt action in such cases. When compared to similar cases, it becomes clear that broad ligament hematomas can result from various obstetric conditions and can present with a range of clinical symptoms. Early diagnosis, surgical exploration, hematoma evacuation, and arterial ligation are critical to prevent negative maternal outcomes.

## References

[1] Saleem N, Ali H S, Irfan A (2009). Broad ligament hematoma following a vaginal delivery in primigravida. Pak J Med Sci.

[2] Kovo M, Eshed I, Malinger G (2006). Broad ligament hematoma following a normal vaginal delivery. Gynecol Surg.

[3] Ziereisen V, Bellens B, Gérard C (2003). Spontaneous rupture of utero-ovarian vessels in postpartal period: a case report and review of the literature. J Gynecol Obstet Biol Reprod (Paris).

[4] Zhang L, Fishman MC, Huang PL (1999). Estrogen mediates the protective effects of pregnancy and chorionic gonadotropin in a mouse model of vascular injury. Arterioscler Thromb Vasc Biol.

[5] Abbas AM, Sheha AM, Ali SS (2017). A rare presentation of ruptured interstitial ectopic pregnancy with broad ligament hematoma: a case report. Middle East Fertil Soc J.

[6] Thanasa E, Thanasa A, Gerokostas EE, Kamaretsos E, Koutalia N, Kontogeorgis G, Thanasas I (2022). Rupture of ectopic ovarian pregnancy accompanied by massive intra-abdominal bleeding and disorder of the coagulation mechanism: a rare and life-threatening obstetric complication. Cureus.

[7] Bhat S, Bhave S (2014). Internal iliac artery ligation: a life saving procedure. J Evol Med Dent Sci.

[8] Di Gennaro D, Damiani GR, Muzzupapa G (2022). Ectopic pregnancy: an overview. Clin Exp Obstet Gynecol.

[9] Nalini N, Singh KA, S N, Kumari A (2023). Clinical profile, risk factors and outcomes of ectopic pregnancy in a tertiary care hospital: a prospective Indian study. Cureus.

[10] Mathesan M, Sharma N (2024). A rare case of broad ligament hematoma following vaginal delivery. Cureus.

[11] Maqbool S, Zulqarnain I, Khan I (2022). Placenta percreta invading left broad ligament in a woman with twin pregnancy: a case report. Ann Med Surg (Lond).

[12] Tokuda H, Nakago S, Kato H, Oishi T, Kotsuji F (2017). Bleeding in the retroperitoneal space under the broad ligament as a result of uterine perforation after dilatation and curettage: report of a case. J Obstet Gynaecol Res.

[13] Kurakula S, Muralidharan V, N N, Kompella AR, K B GB (2023). Incidental finding of a broad ligament hematoma during tubal ligation surgery: a case report and literature review. Cureus.

[14] Ma R, Guan J, Chen J, Sun K, Zhang L, Chen R (2021). Broad ligament pregnancy with pelvic congestion syndrome: a case report. J Obstet Gynaecol Res.

[15] Bankada V, Purra P, Ningappa AM, Jirankal S (2015). A rare case of bilateral broad ligament haematoma in twin pregnancy. J Clin Diagn Res.

[16] Varvoutis M, Nguyen NT, Grotegut C (2021). Spontaneous broad ligament hematoma after vaginal delivery requiring hysterectomy. AJP Rep.

[17] Singh A, Kishore R, Saxena SS (2016). Ligating internal iliac artery: success beyond hesitation. J Obstet Gynaecol India.

[18] Vedantham S, Goodwin SC, McLucas B (1997). Uterine artery embolization - an underused method of controlling haemorrhage. Am J Obstet Gynecol.

[19] Kalburgi EB, NagathaN V, KuNtoji N (2012). Emergency bilateral internal iliac artery ligation - a hospital based cross sectional study. J Clin Diagn Res.

